# Supplementary material for: A cell-free system for mitotically-stimulated TMEJ
Source: bioRxiv. 2026 Oct 28:2026.10.26.762058. Preprint. [Version 1] doi: 10.64898/2026.10.26.762058 (PMC13733922; doi:10.64898/2026.10.26.762058)
Supplement: Supplementary file 1 [file NIHPP2026.10.26.762058V1-supplement-1.pdf]

bioRxiv preprint doi: https://doi.org/10.64898/2026.10.26.762058; this version posted October 28, 2026. The copyright holder for this preprint (which was not certified by peer review) is the author/funder, who has granted bioRxiv a license to display the preprint in perpetuity. It is made available under a CC-BY 4.0 International license.

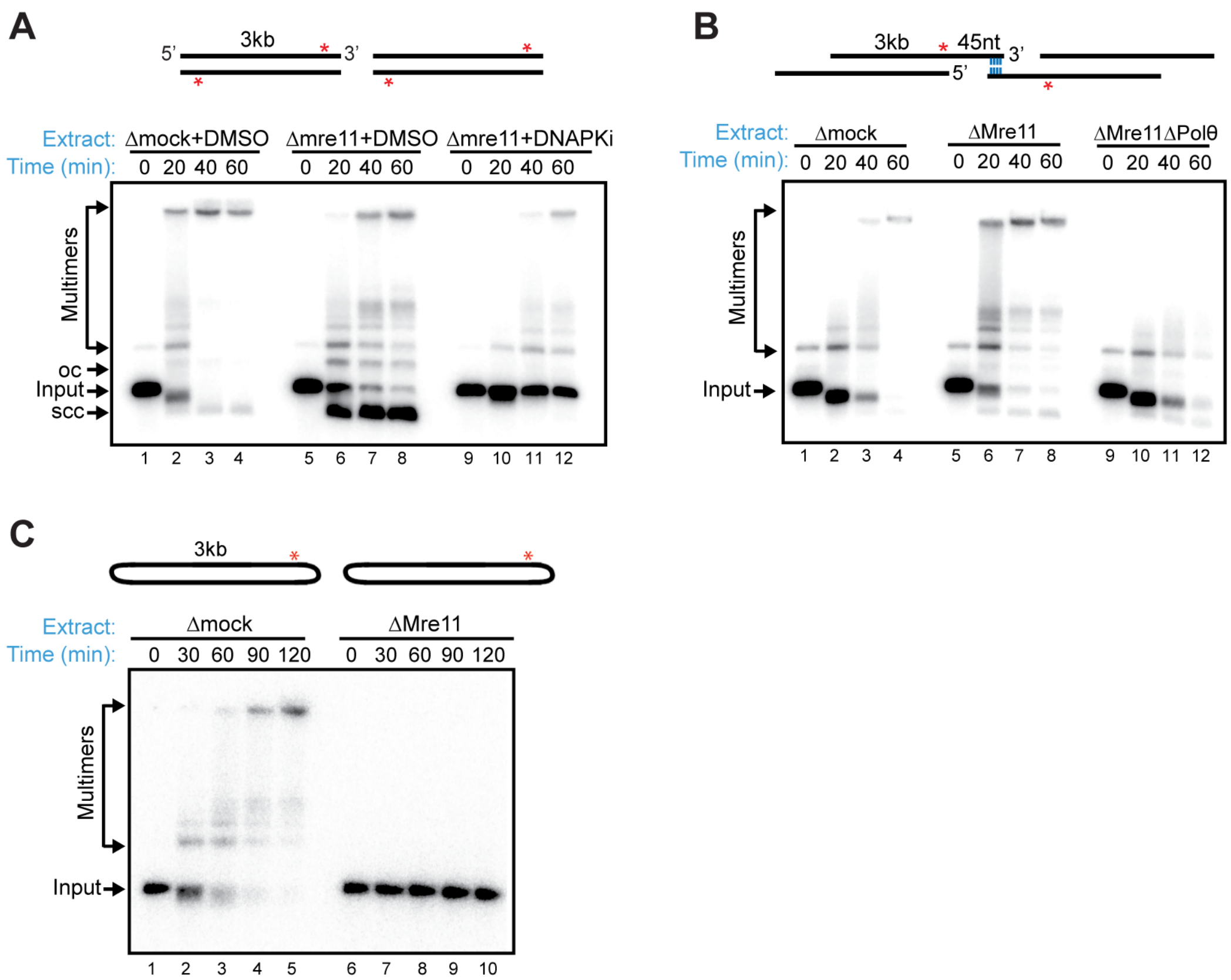

Figure S1. DSB repair pathway choice in *Xenopus* egg extract is dictated by DNA end structures

(A) Linear substrate was incubated with the indicated extracts (optionally supplemented with 10 μM DNA-PK inhibitor NU-7441), separated on a native agarose gel, and subjected to autoradiography. oc, open-circular products; scc, supercoiled closed-circular products. Red star, radiolabel. **(B)** Joining of a linear substrate with 45-nucleotides (nt) 3´ overhangs at both ends in the indicated extracts. Red star, radiolabel. Blue line, terminal microhomology (CCGG-3'). **(C)** Joining of a hairpin substrate in the indicated extracts. Red star, radiolabel.

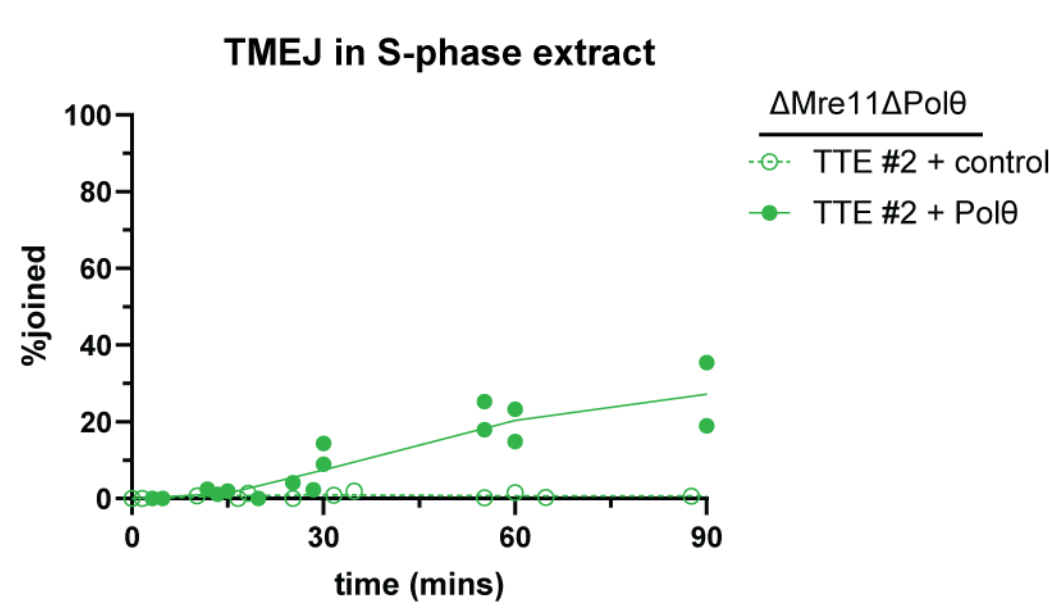

Figure S2. Rescue of TMEJ by recombinant Pol θ in interphase *Xenopus* egg extract

Same as Figure 1E, but using a different lot (#2) of TTE-expressed Pol θ. N=4 experiments. Note that in this case, maximum joining efficiency is ~20%.

bioRxiv preprint doi: https://doi.org/10.64898/2026.10.26.762058; this version posted October 28, 2026. The copyright holder for this preprint (which was not certified by peer review) is the author/funder, who has granted bioRxiv a license to display the preprint in perpetuity. It is made available under a CC-BY 4.0 International license.

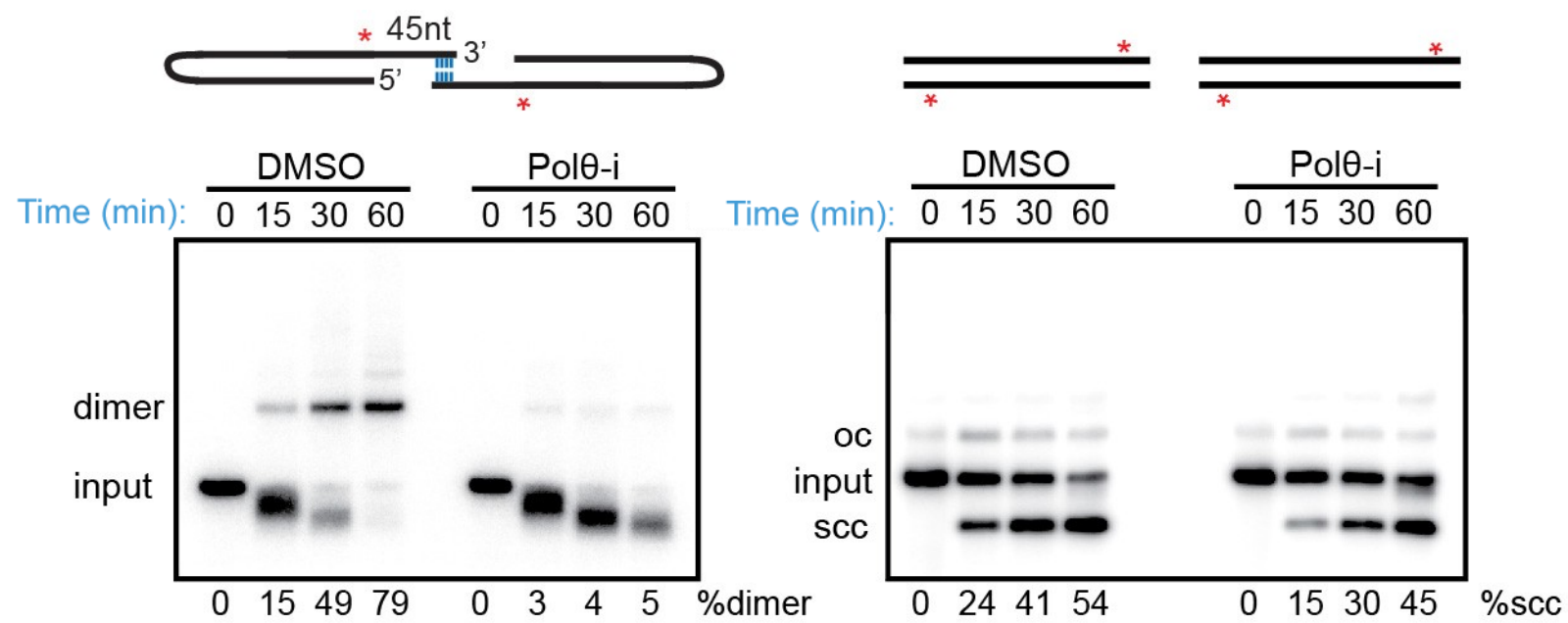

Figure S3. Suppression of TMEJ by a specific Pol θ inhibitor

Joining of the 45OH (left) and the blunt-ended (right) substrates in the presence of DMSO or 10 μM Pol θ inhibitor ART558. oc, open-circular products; scc, supercoiled closed-circular products.

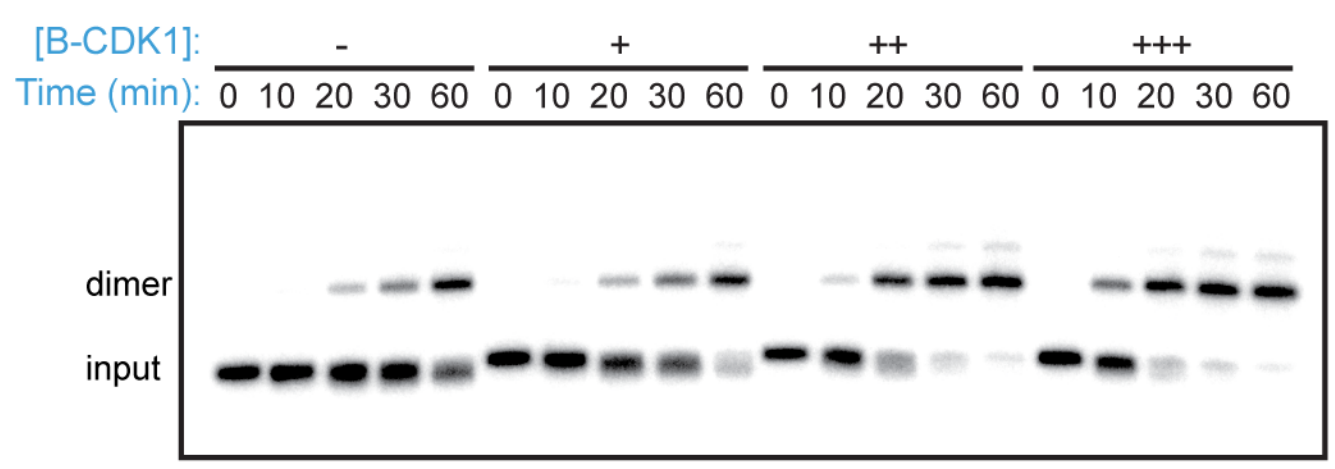

Figure S4. Cyclin B–CDK1 accelerates TMEJ

Non-denaturing gels showing time courses of joining of the 45OH substrate in Mre11-depleted NPE supplemented with increasing amounts of Cyclin B–CDK1 (0, 3, 8.3 and 25 ng/μl).

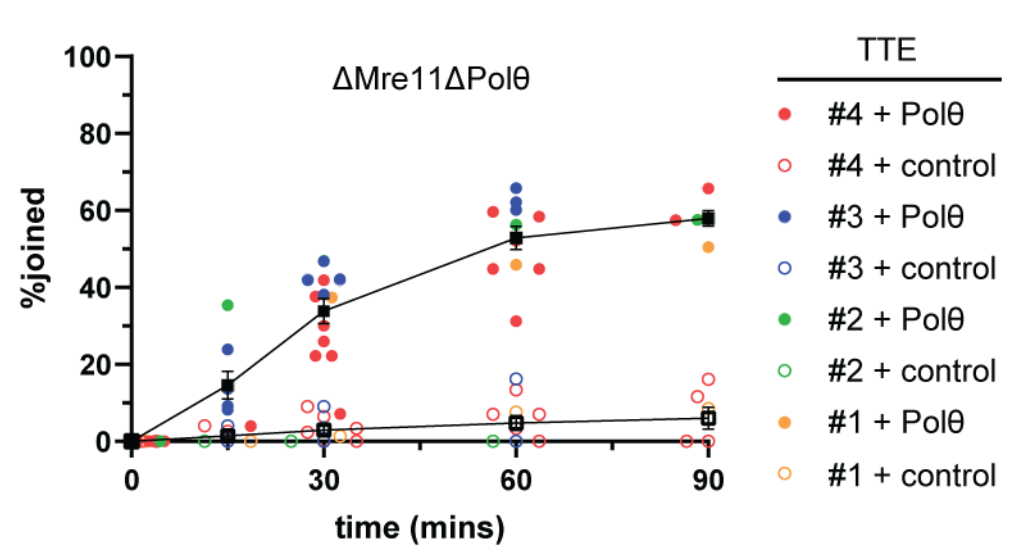

Figure S5. Rescue of TMEJ by recombinant Pol θ in mitotic *Xenopus* egg extract

Quantification of 45OH joining efficiency in NPE depleted of both Mre11 and Pol θ and supplemented with 16 ng/μl Cyclin B–CDK1 and wild-type Pol θ expressed using four different lots of tobacco-cell extract, including those used in Figures 1E and S2. N=13 experiments. Mean ± SEM.

bioRxiv preprint doi: https://doi.org/10.64898/2026.10.26.762058; this version posted October 28, 2026. The copyright holder for this preprint (which was not certified by peer review) is the author/funder, who has granted bioRxiv a license to display the preprint in perpetuity. It is made available under a CC-BY 4.0 International license.

Table S1: Oligonucleotides used to generate DNA substrates

Sequences	Figure (s)
5' GATCAGTGAAGATCCTCACCTTCGGAGTACTCCTTCTGGTTAGAGCCGG	1-3, S2-S5
5' GGCCAGATGTCTTGCGTCCTAGGACGCAAGACATCT	1-3, S2-S5
5' GTCTGGCCTGTCTTAAGTGAAGATCCTCACCTTCGGAGTACTCCTTCTG GTTAGAGCCGG	S1B
5' TAAGACAGGCCAGACCATG	S1B
5' TCTGTCCGTTGCATGAGTGAAGATCCTCACCTTCGGAGTACTCCTTCTG GTTAGAGCCGG	S1B
5' CATGCAACGGACAGAACGT	S1B
5' GATCTGGCCTGTCTTATAAGACAGGCCAGATCCATG	S1C
5' AGATGTCTTGCGTCCTAGGACGCAAGACATCTACGT	S1C
/5Biosg/GG AGA TAG ACA GAT AGG AAA GAT AGA GAG ACA GAG AGA ACG AGA GCG AGG GAG GAG ACA GGA GGA GGA GTG AGA GGC AAA GAG AGG AAA ACA GGA GGA GAG ATA AGA TAC GAG AGA TAG GGA GAG ATG AGG ATG GAC GAG TGA GAG GGC C	3
